# Comparable Exogenous Carbohydrate Oxidation from Lactose or Sucrose during Exercise

**DOI:** 10.1249/MSS.0000000000002426

**Published:** 2020-06-19

**Authors:** OLIVER J. ODELL, TIM PODLOGAR, GARETH A. WALLIS

**Affiliations:** School of Sport, Exercise and Rehabilitation Sciences, College of Life and Environmental Sciences, University of Birmingham, Birmingham, UNITED KINGDOM

**Keywords:** SUGARS, METABOLISM, NUTRITION, SUBSTRATE OXIDATION, PHYSICAL ACTIVITY

## Abstract

**Purpose:**

Ingesting readily oxidized carbohydrates (CHO) such as sucrose during exercise can improve endurance performance. Whether lactose can be utilized as a fuel source during exercise is unknown. The purpose of this study was to investigate the metabolic response to lactose ingestion during exercise, compared with sucrose or water.

**Methods:**

Eleven participants (age, 22 ± 4 yr; V̇O_2peak_, 50.9 ± 4.7 mL·min^−1^·kg^−1^) cycled at 50% *W*_max_ for 150 min on five occasions. Participants ingested CHO beverages (lactose or sucrose; 48 g·h^−1^, 0.8 g·min^−1^) or water throughout exercise. Total substrate and exogenous CHO oxidation was estimated using indirect calorimetry and stable isotope techniques (naturally high ^13^C-abundance CHO ingestion). Naturally low ^13^C-abundance CHO trials were conducted to correct background shifts in breath ^13^CO_2_ production. Venous blood samples were taken to determine plasma glucose, lactate, and nonesterified fatty acid concentrations.

**Results:**

Mean exogenous CHO oxidation rates were comparable with lactose (0.56 ± 0.19 g·min^−1^) and sucrose (0.61 ± 0.10 g·min^−1^; *P* = 0.49) ingestion. Endogenous CHO oxidation contributed less to energy expenditure in lactose (38% ± 14%) versus water (50% ± 11%, *P* = 0.01) and sucrose (50% ± 7%, *P* ≤ 0.05). Fat oxidation was higher in lactose (42% ± 8%) than in sucrose (28% ± 6%; *P* ≤ 0.01); CHO conditions were lower than water (50% ± 11%; *P* ≤ 0.05). Plasma glucose was higher in lactose and sucrose than in water (*P* ≤ 0.01); plasma lactate was higher in sucrose than in water (*P* ≤ 0.01); plasma nonesterified fatty acids were higher in water than in sucrose (*P* ≤ 0.01).

**Conclusions:**

Lactose and sucrose exhibited similar exogenous CHO oxidation rates during exercise at moderate ingestion rates. Compared with sucrose ingestion, lactose resulted in higher fat and lower endogenous CHO oxidation.

Carbohydrate (CHO) is the predominant respiratory substrate at moderate to vigorous exercise intensities (i.e., ≥60% V˙O_2max_) (1). Endogenous CHO (i.e., liver and muscle glycogen) has a finite storage capacity, and depletion of these substrate stores can limit performance in prolonged strenuous exercise (2). Ingestion of CHO during exercise is a well-established nutritional strategy for improving endurance exercise performance and capacity, and operates through sparing endogenous CHO utilization, maintenance of blood glucose concentrations, and total CHO oxidation, and positively affecting the central nervous system (3). CHOs that are known to be readily oxidized, such as glucose, glucose polymers, and combined glucose and fructose or sucrose, form the basis of expert guidelines for CHO feeding during endurance exercise to enhance performance (2,4). Lactose, a CHO found in dairy foods such as milk and yoghurt, does not feature in expert guidelines, and indeed, very little is known about its metabolism in the context of exercise.

Lactose is a disaccharide that consists of a glucose and galactose monomer bound with a β 1–4 glycosidic bond. Galactose has been shown to be oxidized during exercise at ~50% to 60% the rate of glucose (5,6), likely because of the requirement for hepatic metabolism before oxidation. Because of its slow oxidation rate galactose is not generally considered for ingestion during exercise and can result in poorer exercise performance when compared with combined glucose/fructose ingestion (7). Lactose could therefore be less readily available as a fuel source than other CHO owing to the galactose component. However, combined free glucose and galactose ingestion (1:1 ratio) has been shown to improve performance in a ~30-min time trial, completed after 120-min cycling at ~65 V˙O_2max_ to a similar extent to combined glucose and fructose ingestion (80:20 ratio) (7). Furthermore, ingestion of low-fat milk during exercise has been shown to improve time to exhaustion at ~70% V˙O_2peak_ when compared with water ingestion, and to a similar extent to a glucose beverage (8). Milk ingestion does not isolate the effects of the lactose component, but such observations lend support to the idea that lactose may represent a viable energy source during exercise. Indeed, it has been demonstrated that recovery of breath ^13^CO_2_ after ingestion of high ^13^C lactose during very light physical activity (cycling at 50 W) was comparable to glucose (9), perhaps implying a similar oxidation rate. Collectively, the evidence suggests that there could be beneficial metabolic effects of lactose ingestion, but these have not yet been comprehensively studied under exercise conditions representative of moderate to vigorous intensities.

The paucity of research on lactose in an exercise context may, in part, be due to concerns surrounding lactose maldigestion and the potential resulting gastrointestinal symptoms (GIS). GIS during endurance exercise are common, occurring due to mechanical, ischemic, and nutritional factors, and can have severe effects on the performance of athletes (10). GIS can be triggered or worsened by the ingestion of CHO during exercise, especially where ingestion rates exceed absorption rates, and unabsorbed CHO passes into the colon (11). Improvements in exercise performance with CHO ingestion can be dependent on an absence of or minimal GIS (12). Lactose maldigestion, the inefficient or incomplete digestion of lactose, is a concern for some individuals even under resting conditions. Unhydrolyzed lactose reaching the colon may be fermented by colonic bacteria, producing H_2_, CO_2_, CH_4_ and short-chain fatty acids (SCFA), resulting in intolerance symptoms, such as diarrhea, cramping, bloating, and flatulence (13). This lactose intolerance is present in 5%–14% of Caucasians, and presence is higher in other ethnicities (13). However, large boluses of lactose (40–80 g) do not generally trigger symptoms in lactose-tolerant individuals (9), and even in lactose-intolerant individuals, quantities of up to 12–15 g are well tolerated by most (13). Nonetheless, knowledge of GIS would be important in understanding the practical utility of lactose as a CHO source for ingestion during exercise.

The aim of the present study was to compare the exogenous CHO oxidation rates of ingested lactose with sucrose, as a representative readily oxidizable CHO, during endurance exercise and more broadly to characterize the metabolic effects of lactose feeding on exercise metabolism and GIS. The primary hypothesis was that lactose and sucrose would exhibit similar rates of exogenous CHO oxidation during exercise.

## METHODS

### 

#### Participants

Eight men (mean ± SD: age, 23 ± 4 yr; weight, 74.6 ± 10.6 kg; height, 179.7 ± 8.3 cm; V˙O_2peak_, 50.8 ± 5.2 mL·min^−1^·kg^−1^; *W*_max_, 302 ± 48 W) and 3 women (age, 21 ± 3 yr; weight, 55.5 ± 5.5 kg; height, 164.1 ± 3.7 cm; V˙O_2peak_, 51.1 ± 4.0 mL·min^−1^·kg^−1^; *W*_max_, 235 ± 30 W) completed the study. One additional male participant commenced the study but withdrew after experiencing severe GIS in his first experimental trial (ingesting lactose). The sample size was selected to be comparable with previous research that has investigated metabolic responses to CHO ingestion during exercise (12,14–16). Participants were defined as recreationally active and participated in endurance-type exercise at least three times per week, and were included in the study if they attained a V˙O_2peak_ of ≥40 or ≥45 mL·min^−1^·kg^−1^ for female and male participants, respectively. Participants were classified as healthy by successful completion of a general health questionnaire and were excluded if they had a known diagnosis of galactosemia or a history of lactose intolerance. Participants gave written informed consent after having the purpose, risks, and practical details explained to them in accordance with the Declaration of Helsinki. The study was approved by the Science, Technology, Engineering and Mathematics Ethics Committee, University of Birmingham, Birmingham, United Kingdom.

#### Experimental design

Participants completed six visits to the laboratory, including a screening visit and five experimental trials, separated by at least 5 d. Two female participants were using monophasic contraception and completed visits in the active pill consumption phase. One female participant who did not use hormonal contraception self-reported to be regularly menstruating and performed trials in the midfollicular phase of the menstrual cycle (estimated as days 7–10 from menstruation onset) of successive menstrual cycles. All experimental trials consisted of 150 min of exercise on a cycle ergometer at 50% *W*_max_, while ingesting one of five beverages in a randomized order, in a single-blind fashion. The five beverages included lactose with high (LacHi) or low (LacLo) natural abundance of ^13^C stable isotope, sucrose with high (SucHi) or low (SucHi) natural abundance of ^13^C, and water. Venous blood and expired breath samples were collected to characterize substrate oxidation and metabolic responses to CHO ingestion. Breath H_2_ was measured before and after exercise as a proxy for CHO maldigestion (13).

#### Preliminary testing

Participants’ height (Stadiometer Model 220; Seca, Hamburg, Germany) and body mass (Champ II; OHAUSE, Nanikon, Switzerland) were recorded. They then completed a step-incremental exercise test on a cycle ergometer (Lode Excalibur Sport, Groningen, the Netherlands). The test began at 100 W and increased by 30 W every 2 min until volitional exhaustion, or until a cadence of ≥60 rpm could not be maintained. Heart rate (HR) was monitored continuously via radio telemetry (Polar H7, Kempele, Finland). Respiratory gas exchange measurements were performed throughout exercise using an online automated gas analyzer (Vyntus; Vyaire Medical, Mettawa, IL) to determine V˙O_2_ and V˙CO_2_. The highest average 30 s of V̇O_2_ was considered to be V˙O_2peak_. *W*_max_ was calculated as the power output from the final stage completed, combined with the fraction of the time spent in the following stage, multiplied by 30 W, as previously described (17).

Upon completion of the exercise test, participants completed a questionnaire to assess their habitual lactose intake. The questionnaire was based on the 2015 Nurse’s Health Study II questionnaire, Question 28 (18). Alterations were made, including changing units to metric equivalents, removing some listed foods that contain no lactose (sorbet, margarine) or contain negligible amounts (butter) and condensing items with equivalent lactose content into one item (yoghurts). Daily lactose intake was calculated using the weekly or daily frequency of ingestion of each food or drink item, and reference values for the amount of lactose per serving (19,20). Where a range of lactose content was provided, the midpoint of the range was used. Participants habitual lactose intake was 11 ± 10 g·d^−1^, which is congruent with normal lactose intake of 10–12 g·d^−1^ in the Western diet (21,22), with two participants reporting no regular lactose intake.

#### Preexperimental control

Participants were asked to avoid foods with a high natural abundance of ^13^C, to minimize the background shift from glycogen stores for 5 d preceding experimental trials. They were also asked to refrain from alcohol and caffeine, as well as foods that may affect H_2_ breath concentrations (onions, leeks, garlic, cabbage, beans, pickled vegetables, fiber supplements) for 24 h preceding experimental trials. Participants also recorded their diet the day preceding their first experimental trial and replicated it the day before subsequent experimental trials.

#### Experimental trials

Participants attended the laboratory in an overnight fasted state, between 6:00 and 8:30 am. Breath H_2_ concentration was measured in duplicate before and after exercise using a handheld monitor (Hydrogenius; Bedfont Scientific Ltd, Maidstone, England) according to the manufacturer’s instructions, with participants in a seated position. A cannula (Venflon; Becton-Dickinson, Helsingborg, Sweden) was inserted into an antecubital vein and attached to a three-way stopcock (Connecta; Becton-Dickinson) to allow repeated venous blood sampling (LacHi, SucHi, and water trials only). A basal 10-mL blood sample was collected before exercise and every 30 min during exercise, with blood dispensed into an EDTA-containing vacutainer and stored on ice until centrifugation. Resting expired breath samples were collected in duplicate into evacuated 10-mL Exetainer tubes (Labco, High Wycombe, United Kingdom), which were filled from a mixing chamber to determine the ^13^C/^12^C ratio at rest and every 30 min during exercise. The exercise consisted of 150 min of cycling at 50% *W*_max_ (151 ± 24 W for male participants, 118 ± 15 W for female participants) on a cycle ergometer. Respiratory gas exchange measurements (V˙O_2_, V˙CO_2_, and RER) and HR were measured every 30 min during exercise as described previously; RPE values (23) were also taken at these time points. Participants completed a GIS questionnaire at rest and every 30 min during exercise by completing a 100-mm visual analog scale for each symptom. GIS were divided into upper GIS (stomach problems, vomiting, belching, stomach burn, bloating, and stomach cramps), lower GIS (flatulence, urge to defecate, intestinal cramps, diarrhea, and side aches on the left and right), and other symptoms (nausea, dizziness, headache, and urge to urinate) (24). For analysis, an integrated GIS score was derived as the sum of symptoms (in millimeters from visual analog scale) combined between 30 and 150 min for total, upper, lower, and other GIS.

#### Test beverages

Participants ingested a total of 2.4 L of a test beverage in each trial. CHO beverages contained 120 g (w/v) to deliver CHO at a rate of 0.8 g·min^−1^ (48 g·h^−1^) in line with recommendations to provide CHO at 30–60 g·h^−1^ for exercise lasting 2–2.5 h (2). A 600-mL bolus was provided at exercise onset followed by 200-mL drinks every 15 min. To allow quantification of exogenous CHO oxidation, CHOs were selected to have either a high or low natural abundance of ^13^C (LacHi: −16.29 δ‰ vs Pee Dee Bellemnitella (Milk Specialities Global, Eden Prairie, MN); LacLo: −27.73 δ‰ (Volac International, Royston, United Kingdom); SucHi: −11.87 δ‰ (Tate and Lyle, London, United Kingdom); SucLo: −26.13 δ‰ (Aldi, Coventry, United Kingdom). Low ^13^C trials were used exclusively to quantify the background shift in breath ^13^CO_2_ in order to allow for correction and more accurate quantitation of exogenous CHO oxidation.

#### Plasma and breath analyses

Venous blood samples were centrifuged at 1865*g* for 15 min at 4°C. Aliquots of plasma were immediately frozen and stored at −70°C for later analysis. Plasma was analyzed using commercially available kits for glucose, lactate, and nonesterified fatty acid (NEFA) concentrations (Glucose kit, Lactate kit, NEFA kit; Randox, London, United Kingdom) using an automated photometric clinical chemistry analyzer RX Daytona+ (Randox). Plasma galactose concentration was quantified using a colorimetric assay (Sigma Aldrich, St Louis. MO) and insulin using an enzyme-linked immunosorbent assay (Ultrasensitive Insulin ELISA; Mercodia, Uppsala, Sweden).

Isotopic enrichment of breath samples was determined using gas chromatography isotope ratio mass spectrometry (Europa Scientific Hydra 20-20, Crewe, United Kingdom). ^13^C enrichment of ingested CHOs was determined by elemental analyzer isotope ratio mass spectrometry (Europa Scientific Hydra 20-20).

#### Calculations

Fat and total CHO oxidation rates were calculated using the following equations (25), with protein oxidation assumed to be negligible:

$$\text{total}\;\mathrm{CHO}\;\text{oxidation}=4.55\;V˙{\mathrm{CO}}_{2}-3.21\;V˙O_{2}$$

$$\mathrm{fat}\;\text{oxidation}=1.67\;V˙O_{2}-1.67\;V˙{\mathrm{CO}}_{2},$$

where V˙CO_2_ and V˙O_2_ are measured in liters per minute and oxidation rates in grams per minute. Energy expenditure (EE) was calculated using the modified Weir equation (26).

The isotopic enrichment of expired breath samples was expressed as δ per milliliter difference between the ^13^C/^12^C of the sample and a known laboratory reference standard using an established formula (27). δ^13^C was then related to an international standard (Pee Dee Bellemnitella).

The rate of exogenous CHO oxidation was calculated using the following formula:

$$\text{exogenous}\;\mathrm{CHO}\;\text{oxidation}={\mathrm{V\cdot CO}}_{2}\times \left(\frac{\text{δExp}-{\text{δExp}}_{\mathrm{bkg}}}{\text{δIng}-{\text{δExp}}_{\mathrm{bkg}}}\right)\left(\frac{1}{k}\right)$$

where δExp is the ^13^C enrichment of the expired air at various time points in the high ^13^C (LacHi, SucHi) conditions, δIng is the enrichment of the ingested beverage, δExp_bk*g*_ is the ^13^C enrichment of the expired air at the corresponding time points in the low ^13^C (LacLo, SucLo) conditions, and *k* is the amount of CO_2_ (in liters) produced by the complete oxidation of 1 g of glucose (*k* = 0.7467 L). Comparisons of mean and peak exogenous CHO oxidation rates were made only using data from 60 to 150 min during which time recovery of breath ^13^CO_2_ from oxidation approaches 100%, when dilution in the bicarbonate pool becomes negligible (28,29). Endogenous CHO oxidation was calculated by subtracting exogenous CHO oxidation from total CHO oxidation. Subsidiary analyses were also made between different methods of background correction for the calculation of exogenous CHO oxidation as the study design permitted these comparisons. Calculation of exogenous CHO oxidation is often corrected by using the corresponding time points during a water trial (15). In the present study, a low ^13^C CHO condition was used for background correction, and the two methods were compared.

Area under the curve (AUC) was calculated for glucose, lactate, NEFA, and insulin using the trapezoidal method: AUC = (*C*_1_ + *C*_2_/2)(*t*_2_ − *t*_1_), where *C*_1_ and *C*_2_ represent the concentrations of the analyte being calculated, and *t*_2_ and *t*_1_ are the corresponding time points. AUC data are presented as the time-averaged summary value by dividing AUC by the duration of the observation period (i.e., 150 min).

#### Statistical analysis

Experimental data are presented as mean ± SD. Low ^13^C trials were used exclusively to quantify the background shift in breath ^13^CO_2_ and therefore are not included in the statistical analysis. Statistical analysis was performed using SPSS Statistics for Macintosh, Version 25.0 (IBM Corp., Armonk, NY). Differences in mean and peak exogenous CHO oxidation rates between CHO conditions and mean exogenous CHO oxidation using the different background correction methods were compared using paired *t*-tests. Differences between conditions for GIS, HR, RPE, AUC for all plasma analytes, energy, and substrate utilization as a proportion of EE were all analyzed using one-way repeated-measures ANOVA. Mauchly test for sphericity was used, and in cases where this assumption was violated, the Greenhouse–Geisser correction was used. When a main effect was observed, pairwise comparisons were made to locate the source of difference using paired *t*-tests with Bonferroni corrections applied to account for multiple comparisons. Two-way repeated-measures ANOVA was used to analyze differences over time (30–150 min) and between conditions for breath hydrogen, substrate utilization, and plasma metabolites. Where significant interaction (time–condition) effects were detected by ANOVA, *post hoc* pairwise comparisons were made between time points and conditions using paired *t*-tests and the Bonferroni correction to account for multiple comparisons. Statistical significance was set at *P* < 0.05.

## RESULTS

### 

#### Physiological and perceptual characteristics of exercise bouts

As shown in Table 1, there were no significant differences between conditions for either absolute (in liters per minute) or relative (%V˙O_2peak_) oxygen consumption or EE. RER was significantly higher in sucrose than in water (*P* ≤ 0.01) and showed a trend to be lower in lactose than in sucrose (*P* = 0.09) but higher than water (*P* = 0.09). RPE was significantly lower in sucrose than in water (*P* ≤ 0.01) and showed a trend to be lower in lactose than in water (*P* = 0.09). HR was not significantly different between conditions (*P* = 0.17).

**TABLE 1:** Oxygen consumption (absolute and relative), EE, RER, RPE, and HR while ingesting lactose, sucrose, or water, during a 150-min exercise at 50% *W*_max_.

	Lactose	Sucrose	Water
V̇O_2_, L·min^−1^	2.29 ± 0.44	2.20 ± 0.38	2.27 ± 0.40
%V̇O_2_ peak	66 ± 4	63 ± 4	65 ± 4
EE, kcal	1677 ± 330	1607 ± 275	1643 ± 294
RER	0.87 ± 0.03	0.90 ± 0.03*	0.85 ± 0.03
RPE	12.5 ± 1.8	12.1 ± 1.8*	13.4 ± 1.5
HR, bpm	142 ± 4	135 ± 5	140 ± 4

*Significant (*P* < 0.05) difference between sucrose and water.

#### Exogenous and endogenous substrate utilization

**FIGURE 1:**
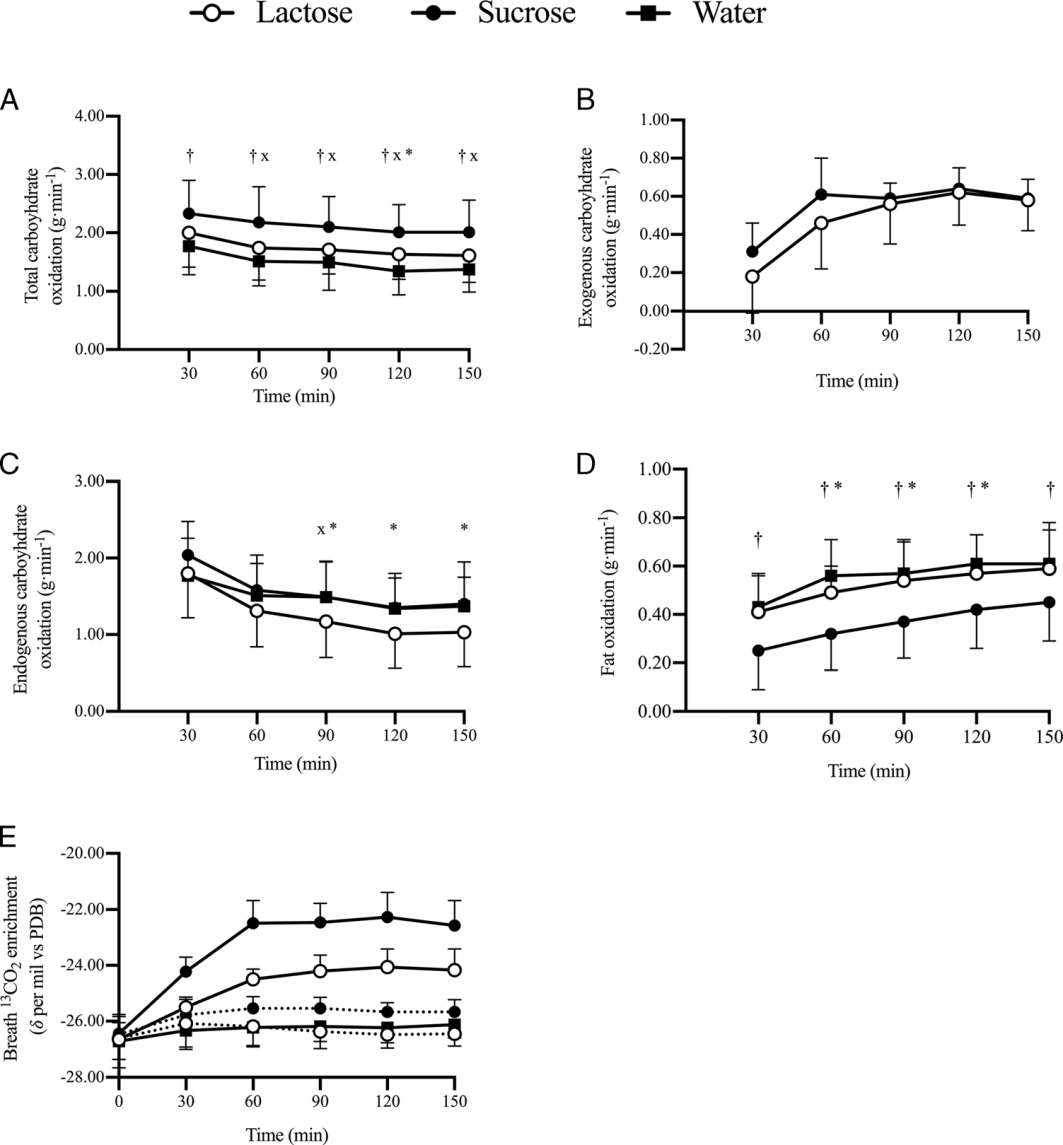
Total CHO (A), exogenous CHO (B), endogenous CHO (C), fat oxidation (D), and change in breath ^13^CO_2_ enrichment (E) between 30 and 150 min of cycling at 50% *W*_max_, ingesting lactose, sucrose, or water, in high ^13^C substrate (―) or low ^13^C substrate (•••) conditions (*n* = 11). *A significant difference (*P* < 0.05) between lactose and sucrose at this time point. ^x^A significant difference (*P* < 0.05) between lactose and water at this time point. †A significant difference (*P* < 0.05) between sucrose and water at this time point.

There was a significant interaction effect (*P* ≤ 0.01; time–condition) in total CHO oxidation (Fig. 1A), and total CHO oxidation was significantly higher in sucrose than in water at all time points (all, *P* ≤ 0.05). Total CHO oxidation was higher in lactose than in water from 60 min onward (all, *P* ≤ 0.05) and higher in sucrose than in lactose at 120 min (*P* ≤ 0.05). Mean exogenous CHO oxidation (between 60 and 150 min; Fig. 1B) was not significantly different between lactose (0.56 ± 0.19 g·min^−1^) and sucrose (0.61 ± 0.10 g·min^−1^; *P* = 0.49). No differences in peak exogenous CHO oxidation between lactose (0.65 ± 0.19 g·min^−1^) and sucrose (0.71 ± 0.13 g·min^−1^; *P* = 0.45) were observed. There were a significant interaction effect (*P* ≤ 0.05; time–condition) in exogenous CHO oxidation and a trend toward higher exogenous CHO oxidation in sucrose (0.31 ± 0.15 g·min^−1^) than in lactose (0.18 ± 0.19 g·min^−1^) at the 30- (*P* = 0.06) and 60-min (*P* = 0.09) time point. There was a significant interaction effect (*P* ≤ 0.05; time–condition) in endogenous CHO oxidation (Fig. 1C), which was significantly lower in lactose than in sucrose at 90 min (*P* ≤ 0.05) and lower in lactose than in water from 90 to 150 min (all, *P* < 0.05). There was a significant interaction effect (*P* ≤ 0.05; time–condition) in fat oxidation (Fig. 1D), which was significantly higher in water than in sucrose from 30 min onward (all, *P* ≤ 0.05) and higher in lactose than in sucrose from 60 to 120 min (all, *P* ≤ 0.05). One subject could not fully complete the water trial because of fatigue and ceased exercise shortly after 120 min, despite completing other trials. Therefore, data from measures of substrate utilization, except exogenous CHO oxidation, were from 0 to 120 min (*n* = 11) and 120 to 150 min (*n* = 10).

**FIGURE 2:**
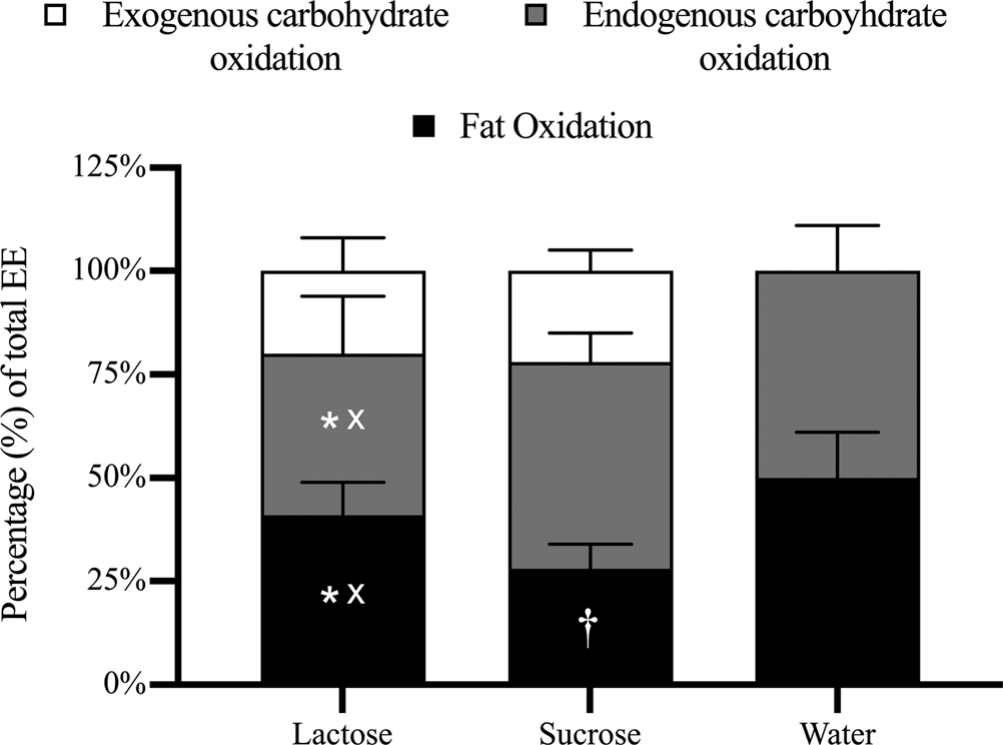
Substrate contributions to total EE from 60 to 150 min. *A significant difference (*P* < 0.05) between lactose and sucrose. ^x^A significant difference (*P* < 0.05) between lactose and water. †A significant difference (*P* < 0.05) between sucrose and water.

Substrate utilization from 60 to 150 min expressed as a proportion of total EE is shown in Figure 2. Exogenous CHO oxidation contributed comparably to EE in both lactose (20% ± 8%) and sucrose (22% ± 5%; *P* = 0.35). Endogenous CHO oxidation contributed least to EE in lactose (38% ± 14%) compared with sucrose (50% ± 7%; *P* ≤ 0.05) and water (50% ± 11%; *P* ≤ 0.01). Fat oxidation contributed most to EE in water (50% ± 11%), greater than both lactose (42% ± 8%, *P* ≤ 0.05) and sucrose (28% ± 8%; *P* ≤ 0.01), which were also significantly different from one another (*P* ≤ 0.01).

Comparison of background correction methods in the calculation of exogenous CHO oxidation rates revealed significantly higher estimation of sucrose oxidation rates using the water correction (0.69 ± 0.15 g·min^−1^) compared with the low ^13^C CHO correction (0.61 ± 0.10 g·min^−1^; *P* ≤ 0.01). Lactose oxidation rates were not significantly different between water-corrected (0.51 ± 0.16 g·min^−1^) and low ^13^C CHO-corrected (0.56 ± 0.19 g·min^−1^; *P* = 0.22). The 95% confidence intervals (i.e., lower, upper) for the mean difference between water-corrected and low ^13^C CHO-corrected exogenous CHO oxidation rates for sucrose and lactose were 0.03 to 0.13 and −0.12 to 0.02 g·min^−1^, respectively.

#### Plasma metabolites

**FIGURE 3:**
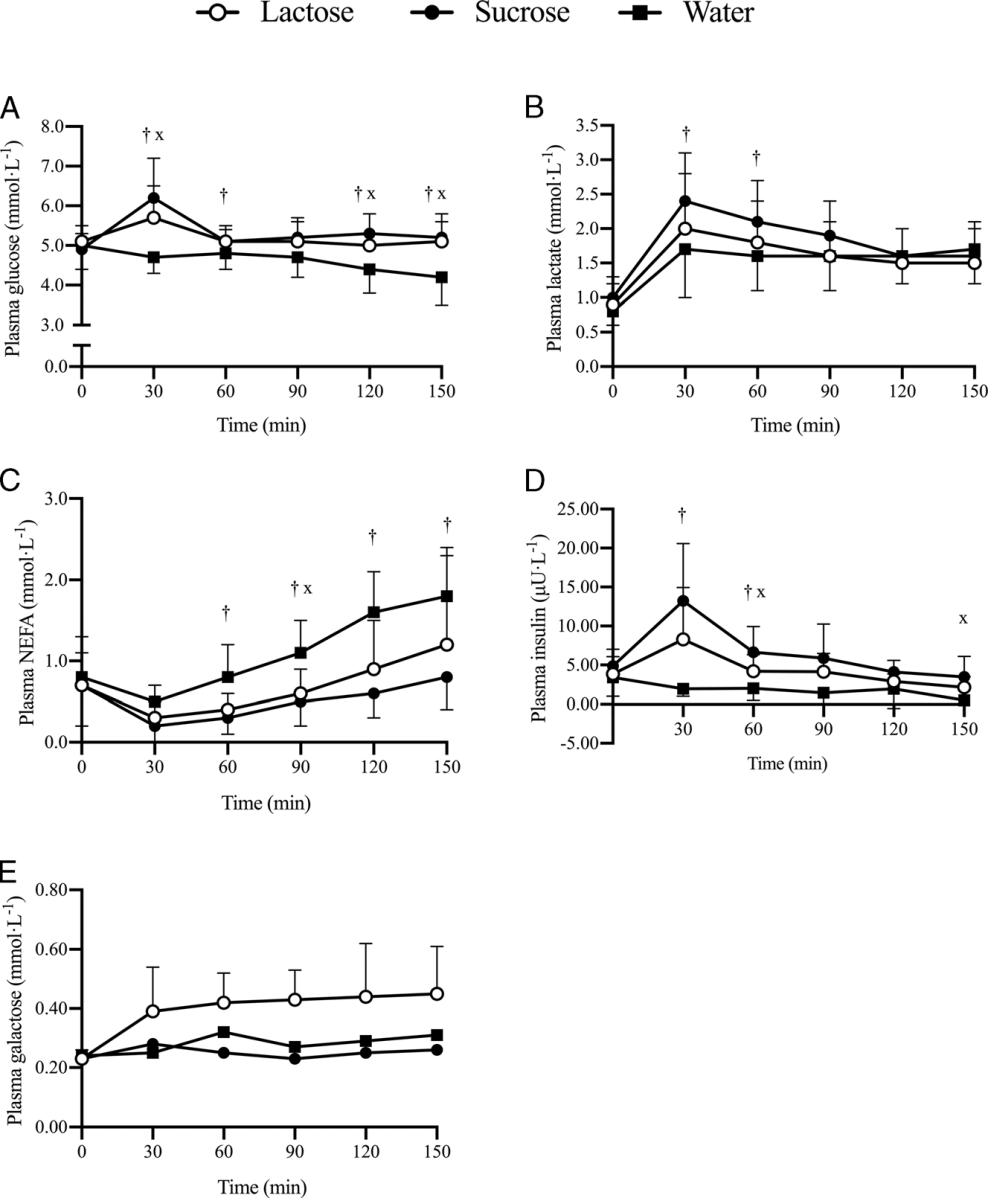
Plasma glucose (A), lactate (B), NEFA (C), insulin (*n* = 8) (D), and galactose (E) concentrations between 30 and 150 min of cycling at 50% *W*_max_, ingesting lactose, sucrose, or water. *A significant difference (*P* < 0.05) between lactose and sucrose at this time point. ^x^A significant difference (*P* < 0.05) between lactose and water at this time point. †A significant difference (*P* < 0.05) between sucrose and water at this time point.

As shown in Figure 3A, there was a significant interaction effect (*P* ≤ 0.01; time–condition) in plasma glucose, which was higher in sucrose than in water from 30 to 60 min and from 120 to 150 min, and higher in lactose than in water at 30 min and from 120 to 150 min. Glucose AUC was significantly higher in lactose (5.2 ± 0.4 mmol·L^−1^, *P* ≤ 0.01) and sucrose (5.4 ± 0.4 mmol·L^−1^, *P* ≤ 0.01) than in water (4.6 ± 0.4 mmol·L^−1^150 min) with no difference between CHOs (*P* = 0.45). There was a significant interaction effect (*P* ≤ 0.05; time–condition) in plasma lactate concentration, which was higher in sucrose than in water from 30 to 60 min (Fig. 3B). There was no main effect of condition (*P* = 0.14) in lactate AUC. There was a significant interaction effect (*P ≤* 0.001; time–condition) in plasma NEFA concentration, which was higher in water than in sucrose than from 60 min onward (all, *P* ≤ 0.05) and higher in water than in lactose at 90 min (Fig. 3C). NEFA AUC was significantly higher in water (1.1 ± 0.3 mmol·L^−1^) than in sucrose (0.5 ± 0.2 mmol·L^−1^, *P* ≤ 0.01) and tended to be higher than lactose (0.7 ± 0.3 mmol·L^−1^, *P* = 0.08), with no difference between CHOs (*P* = 0.62). There was a significant interaction effect (*P* ≤ 0.01; time–condition) in plasma insulin concentration (Fig. 3D), which was significantly higher in sucrose than in water from 30 to 60 min, tended to be higher in lactose than in water (*P* = 0.08) at 30 min, and was significantly higher at 60 and 150 min. Insulin AUC was significantly higher in sucrose (6.8 ± 3.4 μU·L^−1^) than in water (1.9 ± 1.2 μU·L^−1^, *P* ≤ 0.05), which tended to be lower than that in lactose (4.5 ± 2.6 μU·L^−1^, *P* ≤ 0.05), with no significant difference between CHOs (*P* = 0.14). Analysis for plasma galactose was conducted in full in the lactose condition (*n* = 10), as the only condition where galactose was expected to be present, unlike sucrose and water (*n* = 1). Plasma galactose concentrations rose from baseline with lactose ingestion but did not deviate substantially from baseline with sucrose or water ingestion (Fig. 3E). All comparisons of plasma metabolites were *n* = 10, because a cannula could not be placed in the lactose condition in one subject, except insulin, which was *n* = 8, because of limited plasma aliquots.

#### Gastrointestinal discomfort and maldigestion

The maximum scores for upper, lower, and other GIS were 3000, 3000, and 2000 mm, respectively. There were no differences in upper GIS between any conditions (lactose, 12 ± 20 mm; sucrose, 10 ± 22 mm; or water, 10 ± 22 mm; *P* = 0.90), nor were there any differences in lower GIS (lactose, 20 ± 32 mm; sucrose, 29 ± 53 mm; or water, 11 ± 23 mm; *P* = 0.42) or other GIS (lactose, 91 ± 88 mm; sucrose, 104 ± 64 mm; or water, 74 ± 58 mm; *P* = 0.10). Thus, GIS were overall minimal, although one participant experienced severe symptoms, including diarrhea and stomach pain during their first trial (LacHi) and was therefore excluded from the study. One subject experienced milder GIS after the lactose trials but remained in the study.

**FIGURE 4:**
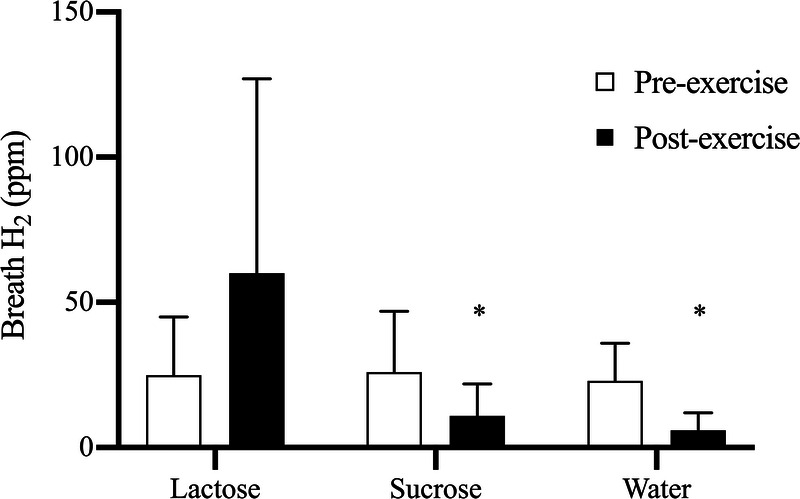
Breath H_2_ concentration (ppm) before and after 150 min of cycling at 50% *W*_max_ and ingestion of lactose, sucrose, or water. *A significant (*P* < 0.05) reduction from preexercise to postexercise (*n* = 11).

There was a significant interaction (time–condition) for breath H_2_ concentration (*P* ≤ 0.05; Fig. 4). Breath H_2_ concentration significantly reduced from preexercise (26 ± 21 ppm) to postexercise in sucrose (11 ± 11 ppm; *P* ≤ 0.01) and water (23 ± 16 to 6 ± 6 ppm; *P* ≤ 0.01). The apparent increase from preexercise (25 ± 20 ppm) to postexercise (60 ± 67 ppm) in the lactose condition was nonsignificant (*P* = 0.14).

## DISCUSSION

The aim of this study was to characterize the metabolic effects of lactose ingestion during endurance exercise as compared with sucrose or water. The primary finding was that mean exogenous CHO oxidation rates from ingested lactose (0.56 ± 0.19 g·min^−1^) were comparable to those of sucrose (0.61 ± 0.10 g·min^−1^) when ingested at moderate rates (<60 g·h^−1^). Furthermore, as compared with sucrose, lactose ingestion resulted in increased fat oxidation and reduced endogenous CHO oxidation, whereas GIS were largely comparable.

The finding that the oxidation rates of ingested lactose and sucrose are similar was in line with the hypothesis. A previous investigation of combined galactose and glucose ingestion during exercise showing similar performance benefits to combined glucose and fructose ingestion would suggest that oxidation of the CHOs could be comparable (7). However, Stannard et al. (7) did not measure exogenous CHO oxidation rates and used free galactose and glucose as opposed to lactose. Stellaard et al. (9) investigated the ingestion of 80 g lactose before light physical activity and showed comparable recovery of breath ^13^CO_2_ after ingestion of high ^13^C glucose or lactose suggesting similar oxidation of the two substrates. It was also shown that the digestion of lactose was not limiting upon its oxidation at this ingestion rate. However, as previously mentioned, the intensity of the protocol (50 W) was very low, and along with the fact that oxidation rates were not directly determined, understanding of the viability of ingested lactose as a fuel source for exercise remained limited. Thus, the present study is the first to show that lactose is readily oxidized during moderate- to vigorous-intensity endurance exercise and is comparable to other typical CHO types when consumed at doses (i.e., 30–60 g·h^−1^) in line with expert guidelines for CHO ingestion during exercise lasting 2–2.5 h (2,30).

The hypothesis in the present study was that lactose and sucrose would be similarly and readily oxidized during exercise. Such a hypothesis may have been questioned given the slow oxidation rate of galactose (~50%–60% the rate of glucose) alone (5,6). O’Hara et al. (31) demonstrated that galactose oxidation was lower than glucose in the first 60 min of a 120-min steady-state cycling protocol but predominated in the second hour, suggesting a delay in galactose oxidation. However, participants in that study ingested a bolus of CHO preexercise, as opposed to regular ingestion during exercise, and therefore, the metabolic effects of the CHO ingestion are not comparable. Nevertheless, the metabolism of galactose is known to be altered when coingested with glucose at rest, such that the presence of glucose more than doubles the first-pass splanchnic clearance of galactose and almost completely ablates the rise in plasma galactose that normally characterizes galactose ingestion (32,33). A blunted rise in plasma galactose has also been demonstrated to occur also with lactose ingestion as compared with galactose-only ingestion, implying that, even with intact lactose, glucose may facilitate galactose metabolism (32). The oxidation rates of lactose during exercise suggest that galactose metabolism has been augmented such that lactose and sucrose were oxidized to a comparable extent. However, whether glucose facilitates galactose metabolism remains to be tested in an exercise context. Regardless, the oxidation rates of both lactose and sucrose observed herein are entirely consistent with those observed previously with glucose or glucose–fructose ingestion at similar doses (0.8 g·min^−1^), exercise intensity (55% *W*_max_), and duration (150 min) (30), further supporting the potential of lactose as a readily oxidizable fuel substrate.

Digestion of lactose is limited in many individuals at rest because of low activity or absence of lactase (13) and was therefore a potential concern in the present study. Indeed, there was a trend toward higher sucrose oxidation in the first 60 min, which could suggest a delay in lactose digestion. It is possible that this may be attributable to the higher *K*_m_ of sucrase (~142 mM) than lactase (~14 mM) (34,35), which at the very least implies that the scope for increasing above-moderate ingestion rates is greater in sucrose than in lactose. Nonetheless, there is methodological uncertainty surrounding the saturation of the bicarbonate pool in the early stages of exercise (28,29), and oxidation rates of lactose and sucrose were not ultimately different as exercise duration progressed. This suggests that any potential digestion limitation with lactose at the ingestion rates used herein was transient in respect of the overall effect on exogenous CHO oxidation.

There were markedly different responses of breath H_2_ to lactose ingestion, with five subjects experiencing a reduction from preexercise to postexercise, whereas the remaining six increased breath H_2_. By comparison, breath H_2_ reduced significantly from preexercise to postexercise in both sucrose and water conditions. Although the increase in breath H_2_ with lactose ingestion was not significant, it suggests that digestion was incomplete and fermentation of lactose in the colon occurred in some individuals. Indeed, both subjects who experienced GIS markedly increased breath H_2_, although some nonsymptomatic subjects increased breath H_2_ to a similar or greater extent. This variable response of breath H_2_ suggests that its measurement may not be an accurate proxy for symptomatic CHO maldigestion during exercise. It is possible that some of the recovered breath ^13^CO_2_ after lactose ingestion may be of colonic origin, from fermentation of lactose by bacteria such as *Bifidobacteria* (36). Fermentation may produce CO_2_, which is absorbed into the circulation and expired, and would be detected as breath ^13^CO_2_. It is possible that this could lead to slight overestimation of rates of exogenous CHO oxidation of lactose, although the extent to which this occurred in the present study cannot be determined, and the quantities of CO_2_ arising from fermentation are not known. In addition, fermentation may have resulted in the production of SCFA, which could be oxidized during exercise and increase recovery of breath ^13^CO_2_. Regardless, lactose ingestion resulted in physiological bioavailability, evidenced by increase in plasma glucose, galactose, and insulin. Importantly, the beneficial changes in plasma glucose are comparable to sucrose, which is an important and expected outcome of effective CHO supplementation in exercise and may suggest an at least similar potential for ergogenic effects of lactose and sucrose.

Given the importance of gut comfort for exercise performance (11) and the presence of lactose intolerance in some individuals, investigation of GIS was particularly important. Upper, lower, and other GIS were predominantly mild, with no differences between conditions. However, two subjects experienced noteworthy symptoms with lactose ingestion. One experienced severe lactose intolerance symptoms during exercise (flatulence, urge to vomit, and diarrhea) and was excluded from the study. The other participant experienced similar but milder symptoms in the hours after exercise. This participant demonstrated a discrepancy in mean exogenous CHO oxidation rates between lactose (0.34 g·min^−1^) and sucrose (0.61 g·min^−1^). Both subjects reported regular dairy and lactose ingestion. In another participant, a marked disparity in mean exogenous CHO oxidation between lactose (0.15 g·min^−1^) and sucrose (0.73 g·min^−1^) was observed alongside large increases in breath H_2_ (17–208 ppm) with lactose, yet GIS were mild and comparable in both conditions. Collectively, these preliminary observations are interpreted to mean that GIS with the moderate dose lactose feeding protocol used herein are unlikely to be problematic for most individuals, although caution should be taken as some apparently lactose-tolerant individuals may experience symptoms.

Higher fat oxidation and lower estimated endogenous CHO oxidation were observed with lactose ingestion than sucrose ingestion. Fat oxidation is known to be suppressed with CHO ingestion during exercise, which in part occurs because of suppression of lipolysis by insulin, which reduces plasma free fatty acids (37). However, there is also a direct effect of CHO ingestion and resulting insulinemia on muscle, which suppresses mitochondrial long-chain fatty acid oxidation (38). Although plasma insulin levels were not significantly different between the CHO conditions, insulinemia was greater at 30 min in sucrose than in water, but not in lactose. There is also an evident visual disparity in insulin concentrations and AUC between sucrose and lactose. Lactose is known to be less insulinogenic than CHOs such as glucose at rest (39), and this may extend into exercise. It is possible that the higher insulin levels in the sucrose condition suppressed fat oxidation, therefore increasing reliance on endogenous CHO oxidation, whereas an attenuated insulin response with lactose ingestion did not trigger such extensive suppression of fat oxidation. The presence of plasma lactate has also been demonstrated to reduce lipolysis during exercise (40). Higher concentrations of lactate were present in the sucrose condition than in water, which may contribute to the lower fat oxidation observed in the sucrose condition. The maintenance of high fat oxidation rates with lactose ingestion may have permitted the lower endogenous CHO oxidation with lactose ingestion versus sucrose, which represents glycogen sparing. Because CHO (both muscle and liver glycogen) is the most important substrate in moderate to vigorous exercise intensities, glycogen depletion can contribute to poorer endurance exercise performance, and therefore, reducing reliance on endogenous CHO oxidation may benefit exercise performance (3,41,42). However, the observed sparing of endogenous CHO with lactose ingestion versus water ingestion is of unclear origin and necessitates further investigation.

Finally, because of the design of the present study, it was possible to perform a subsidiary analysis to compare different methods of background correction in the calculation of exogenous CHO oxidation. Using a water trial for correction is commonplace to account for the background shift in breath ^13^CO_2_/^12^CO_2_ that occurs during exercise, which is attributable to the oxidation of residual ^13^C in endogenous CHO stores. The present study demonstrated that a water correction significantly overestimated exogenous CHO oxidation of sucrose by approximately 15% and underestimated lactose oxidation rates by 10%, although nonsignificantly. Ingesting the corresponding CHO with low natural abundance of ^13^C for each CHO accounts for the sugar-specific effects of CHO ingestion on metabolism and the subsequent shift in breath ^13^CO_2_, which a water correction fails to do. Furthermore, using a water correction increases the possibility of participants fatiguing in the water trial because of a lack of CHO, which occurred to one participant in the present study. Using a low ^13^C CHO correction reduces the possibility of fatigue due to a lack of CHO in background correction trials. If a water correction had been utilized in the present study, a difference in exogenous CHO oxidation rates between the sugars would have been concluded because of the significant overestimation of sucrose oxidation. The absence of a clear influence of background correction method on calculated lactose oxidation rates may be attributable to the effects of lactose ingestion on the overall metabolic response being less pronounced than those of sucrose. These findings suggest that past studies using a water correction and naturally high-abundance ^13^C CHOs may have, depending on the nature of the CHOs investigated, overestimated exogenous CHO oxidation rates. Future investigations utilizing naturally high-abundance ^13^C CHOs should consider using a CHO-specific low ^13^C background trial to more accurately quantify exogenous CHO oxidation.

In summary, the present study demonstrated that lactose ingested during exercise is oxidized at a comparable rate to sucrose and maintains plasma glucose similarly at moderate ingestion rates. Furthermore, lactose ingestion reduced endogenous CHO oxidation and increased fat oxidation relative to sucrose ingestion. Future investigations could seek to determine the source of endogenous CHO sparing (i.e., liver and/or muscle glycogen), the potential for lactose ingestion at higher rates, and whether lactose ingestion during exercise can beneficially influence exercise performance.
